# CT Attenuation of Hepatic Pancreatic Cancer Metastases Correlates with Prognostically Detrimental Metastatic Necrosis

**DOI:** 10.3390/jcm12237319

**Published:** 2023-11-26

**Authors:** Stefan Reischl, Sebastian Ziegelmayer, Markus Graf, Joshua Gawlitza, Andreas Philipp Sauter, Manuel Steinhardt, Marie-Christin Weber, Philipp-Alexander Neumann, Marcus Richard Makowski, Fabian Karl Lohöfer, Carolin Mogler, Rickmer Früdd Braren

**Affiliations:** 1 Institute of Diagnostic and Interventional Radiology, School of Medicine and Health, Technical University of Munich, 81675 Munich, Germanymanuel.steinhardt@tum.de (M.S.); rbraren@tum.de (R.F.B.); 2 Department of Surgery, School of Medicine and Health, Technical University of Munich, 81675 Munich, Germanyphilipp-alexander.neumann@tum.de (P.-A.N.); 3 Institute of Pathology, School of Medicine and Health, Technical University of Munich, 81675 Munich, Germany; carolin.mogler@tum.de; 4 German Cancer Consortium (DKTK, Partner Site Munich), German Cancer Research Center (DKFZ), 69120 Heidelberg, Germany

**Keywords:** pancreatic cancer, necrosis, imaging, computed tomography, biopsy, intervention

## Abstract

Percutaneous CT-guided biopsy is a frequently performed procedure for the confirmation and molecular workup of hepatic metastases of pancreatic ductal adenocarcinoma (PDAC). Tumor necrosis of primary PDAC has shown a negative prognostic impact in recent studies. This study aims to examine predictability in CT scans and the prognostic impact of necrosis in hepatic metastases of PDAC. In this tertiary-center retrospective cohort study, we included 36 patients with hepatic metastases of PDAC who underwent CT-guided hepatic biopsies. Normalized attenuation of the biopsied metastasis was determined in venous phase contrast-enhanced planning scans obtained prior to biopsy by automatic, threshold-based 3D segmentation and manual, blinded 2D segmentation. A board-certified pathologist specialized in hepatic pathology histologically quantified the tumor necrosis and cellularity of the biopsy cylinders. We found a significant inverse-linear correlation between normalized attenuation and the fraction of necrosis (Pearson’s r = 0.51, *p* < 0.001 for automatic 3D segmentation or Pearson’s r = 0.52, *p* < 0.001 for manual 2D segmentation), whereas no correlation was found with tumor cellularity. Additionally, we discovered that patients with a fraction of necrosis ≥ 20% in metastases had a significantly shorter overall survival (*p* < 0.035). In summary, tumor necrosis of PDAC metastases can be estimated from contrast-enhanced CT scans, which could help to improve biopsy sample pattern planning. In addition, liver metastatic necrosis may serve as a prognostic biomarker in PDAC.

## 1. Introduction

Despite extensive scientific effort during recent years, pancreatic ductal adenocarcinoma (PDAC) remains one of the tumor entities with the poorest prognosis, with a 5-year survival rate of less than 10% [1]. PDAC is by far the most common malignant primary tumor of the pancreas and the second most frequent malignant tumor of the digestive system [2]. Although resection can be attempted in the nonmetastatic stage, and neoadjuvant systemic therapy can potentially precede surgery in borderline-resectable situations, disease is usually not curable. In case of metastatic PDAC palliative chemotherapy is the cornerstone of systemic therapy.

According to current German guidelines [3], prior to initiation of the palliative systemic therapy of suspected metastatic PDAC, histopathologic confirmation should be pursued. Of the available options, endosonographic fine needle biopsy and percutaneous CT- or ultrasound (US)-guided tru-cut biopsy, no procedure is preferred by the guidelines, but the approach with the least risk and highest chance of success should be selected. Although basically equally suitable, the decision between the different percutaneous methods (US- or CT-guided) may vary between the centers and be driven by expertise and individual preferences. In case of CT-guided liver biopsy, in many cases, more than one potential lesion is technically accessible. For robust morphomolecular (e.g., cancer genome sequencing) workup, biopsy material should be collected from vital tumor regions to avoid cellular damage and supply a sufficient amount of material. Thus, to potentially improve sampling patterns, determining whether cellularity and necrosis of metastases can be estimated via CT imaging was a central motivation of this project.

Beyond this, tumor tissue composition and its representation in noninvasive imaging-based tumor characterization has been the subject of intense research over recent years. In a systematic review, the presence of tumor necrosis of solid tumors was reported for several entities, including breast, renal cell, non-small cell lung, and colorectal cancer, as a negative prognostic histopathological feature [4]. In the case of PDAC, a recent meta-analysis identified the detrimental prognostic role of tumor necrosis of the primary tumor in histopathology on overall survival (OS), recurrence-free survival, vascular and neural invasion, T stage, M stage, and grading [5,6,7,8]. Another recent study described a significant correlation of MRI-depicted tumor necrosis of PDAC to tumor size, nodal status, and metastases [9], while another study reported higher tumor grading and higher cellularity co-occurring with tumor necrosis determined by MRI [10]. However, the presence of tumor necrosis in relation to PDAC metastases has not been reported on thus far.

Tumor cellularity was also shown to be characteristic of more aggressive subtypes of PDAC in various studies [11,12,13,14], with patients containing high cellularity regions in the primary tumor of PDAC experiencing a significantly shorter OS [14].

Thus, if predicted by imaging, necrosis and cellularity of PDAC metastases could be valuable prognostic biomarkers. Previous studies have shown an inverse relationship between the attenuation and tumor cellularity of primary PDAC in contrast-enhanced CT scans [14].

This study aimed to correlate the attenuation of hepatic PDAC metastases in contrast-enhanced CT scans acquired prior to percutaneous biopsies with the histological presence of necrosis and cellularity. Furthermore, we aimed to validate tumor necrosis of metastases as a negative prognostic biomarker in metastatic PDAC. Our findings could have important implications for biopsy pattern planning and oncologic prognosis estimation.

## 2. Materials and Methods

### 2.1. Patient Inclusion

This study was designed as single-center, retrospective cohort study at our tertiary institution. It was approved by the ethical review board of our institution (No. 180/17S) and was performed in accordance with the Declaration of Helsinki. Informed consent was waived according to the regulations of our institution for retrospective analyses. A Picture Archiving and Communication System (PACS) query was performed via the following search term strategy: ((‘pancreatic’ and (‘cancer’ or ‘adenocarcinoma’ or ‘carcinoma’)) or ‘PDAC’) and ’biopsy’. Subsequently, a stepwise exclusion approach was performed as follows: (1) exclusion of all cases with nonhepatic biopsies, (2) exclusion of all cases with a histopathological report not consistent with PDAC metastases, and (3) exclusion of all cases with insufficient image quality (e.g., major motion artifacts/noncontrast-enhanced scans).

### 2.2. CT-Guided Biopsy Procedure

Image acquisition and biopsy were performed on a Philips IQon Spectral CT scanner or a Philips Brilliance iCT 256 CT scanner (Philips Healthcare, Best, The Netherlands). All included cases underwent a contrast-enhanced diagnostic CT scan. Weight-adapted doses of Iomeprol (Bracco S.p.A., Milan, Italy) followed by a saline chaser of 20 mL were administered as i.v. contrast agent 70 s before the diagnostic scan of the whole liver volume in venous phase. All included biopsies were performed according to our standard procedure: Biopsy planning was performed in the diagnostic scan. Stacks of three adjacent axial images of 3 mm slice thickness were acquired throughout the procedure for manual navigation of the needle tip until biopsies were retrieved at the target position with a BARD magnum biopsy system (BD, Franklin Lakes, NJ, USA) at a penetration depth of 22 mm with a 17/18 G coaxial needle system. Biopsy cylinders were immediately fixated in 10% formaldehyde and transferred to the Institute of Pathology for further histopathologic workup.

### 2.3. Automatic and Manual Image Segmentation

**Figure 1:**
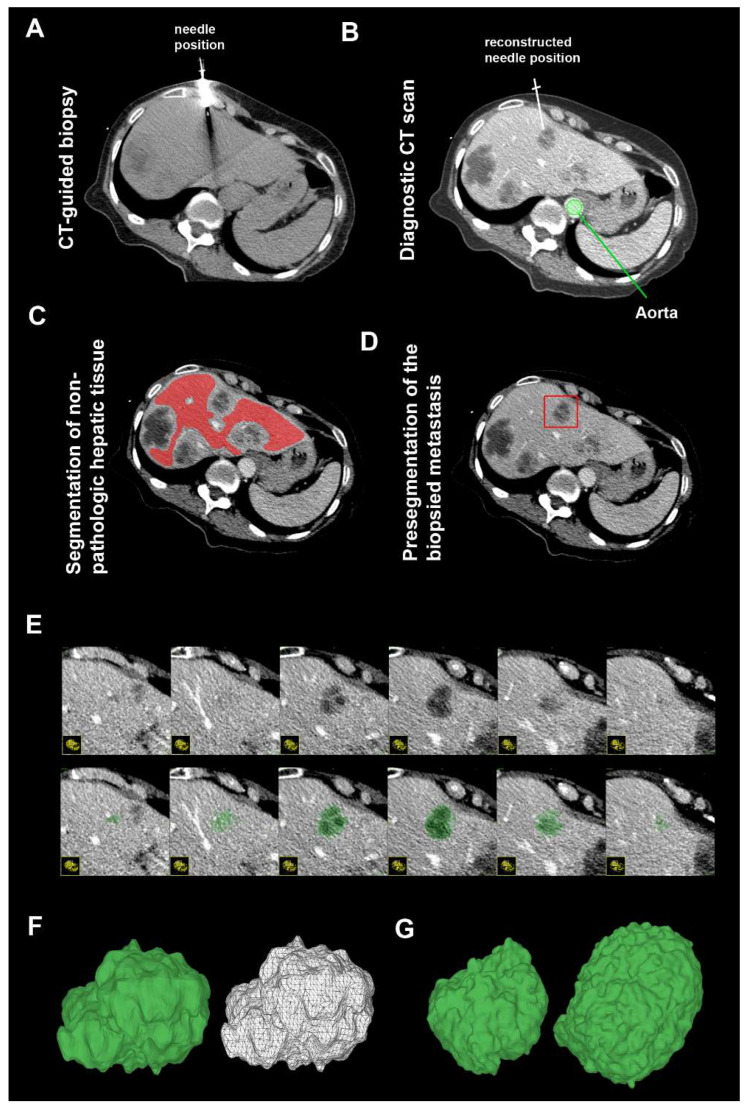
Automatic two-sided threshold-based 3D segmentation of biopsied metastasis. Diagnostic CT scans (axial slices; slice thickness 0.9 mm; isotropic voxels) acquired in the venous phase (70 s after i.v. injection of iodine-based contrast agent) were analyzed retrospectively. (**A**) The most recent position of the biopsy needle in the images before acquisition of biopsy cylinders was used as the reference position for further analyses. (**B**) In the corresponding slice of the diagnostic CT scan, the biopsied metastasis was identified, and the attenuation of contrast-enhanced aortic blood pool was determined with a circular ROI. (**C**) Nonpathologic hepatic tissue was segmented, with vessels and other metastases being avoided, to determine the average attenuation of normal liver tissue. (**D**) Presegmentation of the metastasis was performed with a 3D bounding box. An initializing bubble was positioned centrally. (**E**) Automatic 3D segmentation in the two-sided threshold mode (upper threshold: attenuation of nonpathologic hepatic tissue—10 HU) resulted in a standardized 3D full-volume segmentation of the lesion. (**F**) 3D volume-rendered surface mesh of the exemplary metastasis (from (**A**–**E**)) and two further exemplary patients (**G**) are visualized.

Retrospective image analysis was performed with the aim to determine the origin of the biopsy cylinders. Automatic 3D segmentation of the biopsied metastasis was performed using ITK-SNAP [15] in axial slices (slice thickness 0.9 mm, isotropic voxels; see Figure 1). In detail, the last position of the needle immediately before biopsy was determined in the planning scan by review of the pedal-acquired images. Nonpathologic liver tissue, with metastases and blood vessels being avoided, was manually segmented in this slice to determine the mean attenuation of normal liver tissue, which served as upper threshold for automatic segmentation (shown in Figure 1C). The biopsied metastasis was presegmented with a 3D bounding box with a safety margin around the lesion (demonstrated in Figure 1D) prior to automatic segmentation. Automatic 3D segmentation was performed in the two-sided threshold mode. The lower threshold was set to −20 HU. The upper threshold was calculated as follows:

$$Upper\;threshold\left(in\;HU\right)=attenuation\;of\;non-pathologic\;liver\;tissue\left(in\;HU\right)-10\;HU.$$

One initializing bubble was placed centrally within the biopsied metastasis and autosegmentation was run for 1000 iterations under the following parameters: region competition force = 1.0, smoothing force = 0.2, speed-up factor 1.0, and step size = 1. This resulted in a standardized, automatic 3D segmentation of the full volume of the metastasis (Figure 1E). In a few cases, manual correction of the segmented volume had to be performed in the case of autosegmentation of extrahepatic tissue. Surface meshes of the 3D-segmented metastases were visualized via volume rendering in 3D Viewer “https://3dviewer.net (accessed on 14 October 2022)” (Figure 1F,G). Additionally, a polygonal, freehand ROI of the whole metastasis was drawn manually in the single slice of the last needle position to determine its attenuation, as this is the most feasible approach in clinical routine. Manual segmentation was performed by a resident radiologist who was blinded to the remainder of patient data. An additional circular ROI was drawn in the aorta on the same slice to measure the attenuation of the contrast-enhanced blood pool in the aorta for normalization, as described previously [14]. The corresponding attenuation of the (either automatic 3D or manual 2D segmentation) metastasis was normalized to the attenuation of the aorta as follows:

$$normalized\;attenuation={attenation}_{Metastasis}/{attenuation}_{Aorta}$$

### 2.4. Histopathological Data

Histologic slices were retrieved from the biobank of the Institute of Pathology of our tertiary center. Hematoxylin-and-eosin-stained slices of the biopsy cylinders processed in routine histopathologic workup earlier were collected. One representative slide per patient was scored quantitatively by a board-certified pathologist specialized in hepatic pathology. The following items were scored: the percentage of malignant tissue of total tissue; the percentage of necrotic tissue of total malignant tissue; the cellularity within the malignant, nonnecrotic tissue (low: <33%; intermediate: 33–67%; high: >67%); and the number of biopsy cylinders/fragments. The methodology regarding pathologic quantification was in accordance with but more precise than that of the previous literature, in which necrosis was measured quantitatively in primary PDAC and defined as “confluent cell death in invasive areas of primary cancers” [6,7,8]. In two studies, the classification was “absent/present” [7,8], while in one study, subclassification was “large/small” [6]. Our study is the first to analyze tumor necrosis of metastases in general with additional quantification in percentages. The cellularity of primary PDAC has been measured in several previous preclinical and clinical studies [12,13,14]. We scored tumor cellularity according to these studies, which reported quantification by one or two experienced pathologists into two or three classes (<30%/>30% [13], <30%, 30–70%, >70% [12,14]).

### 2.5. Survival Analyses

Kaplan–Meier survival analysis was employed to estimate the OS probabilities for three size-balanced metastasis tumor necrosis categories (high: ≥20% necrosis; intermediate: ≥5% to <20% necrosis; low: <5% necrosis). All patients that received any type of systemic therapy prior to the included biopsy or that had metachronous metastases were excluded, as OS timespan could not be determined, which led to the further exclusion of 5 patients. The log-rank test was used to assess the statistical significance of differences in survival curves between these categories. Pairwise comparisons were performed between the groups.

### 2.6. Statistics

All statistical analyses were performed using SPSS (Version 28, IBM, Armonk, NY, USA). Data visualization was performed in GraphPad Prism (Version 9, GraphPad software Inc., San Diego, CA, USA). Parametric data are presented as the mean ± standard deviation. Normal distribution was tested for with the Shapiro–Wilk test. Statistical differences between normally distributed parametrical variables were determined using one-way ANOVA. Pearson’s correlation was performed for the analysis of linear correlation between parametric variables.

## 3. Results

### 3.1. Inclusion Process and Study Collective

**Figure 2:**
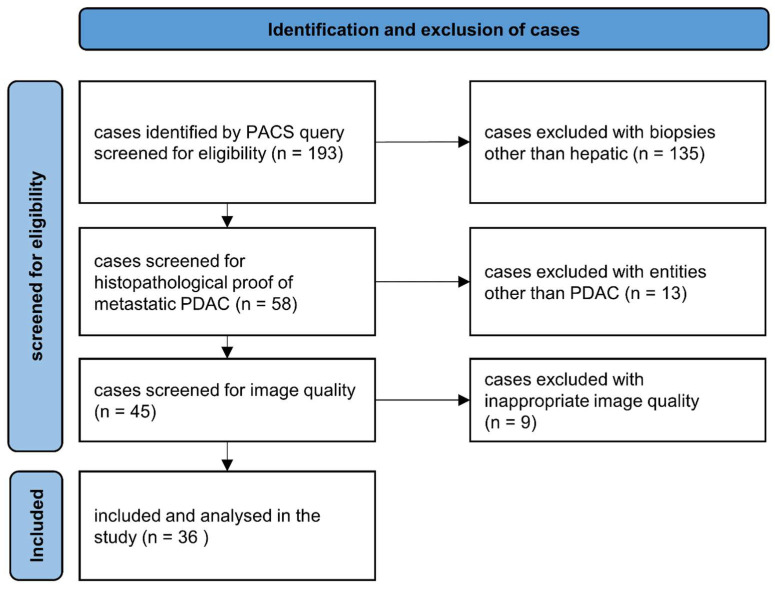
Patient inclusion flowchart. Eligible cases were identified via a PACS query. After multiple exclusion steps, cases with CT-guided biopsies of proven hepatic metastases of PDAC were included and analyzed in the study.

Overall, 193 patients were identified with the PACS search query and screened for eligibility. Of these 193 patients, 135 cases were excluded for having nonhepatic biopsies. Another 13 patients were excluded for being diagnosed with entities other than metastatic PDAC (e.g., primary liver tumors, metastasis of different primary tumors). An additional nine patients were excluded for inappropriate image quality (e.g., noncontrast-enhanced imaging) (Figure 2).

In total, 36 patients with CT-guided biopsies of histopathologically proven hepatic metastases of PDAC between September 2009 and January 2021 were included and analyzed in the study. These patients had a mean age of 65.5 ± 10.5 years. The enrolled group consisted of 19 (53%) females and 17 (47%) males. In 4 of the 36 included cases, the hepatic metastases were metachronous metastases after resection of the primary tumor. Of these, two cases were resected without adjuvant chemotherapy prior to the biopsy. Only two patients received systemic chemotherapy prior to the included CT scan (which ended 18 months and 6 years prior, respectively). The patient group had a mean normalized attenuation of automatic 3D-segmented metastases of 0.45 ± 0.11, while that for manually 2D-segmented metastases was 0.46 ± 0.14. A summary of the clinical and histopathological characteristics of all included patients is shown in Table 1.

### 3.2. Pathologic Quantification of Necrosis and Cellularity of the Biopsy Cylinders

**Figure 3:**
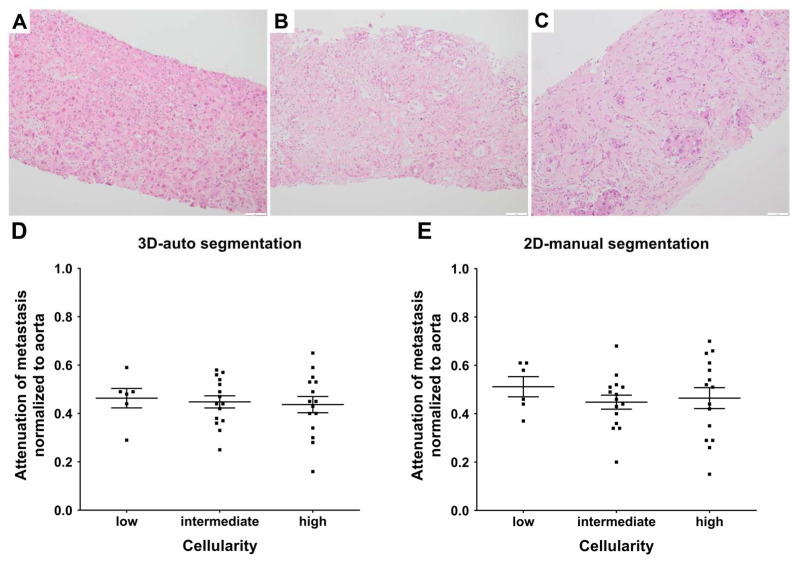
Correlation of attenuation with the cellularity of PDAC metastasis. Attenuation in Hounsfield Units (HU) of the automatic 3D-segmented and the manual, single-slice, 2D-segmented biopsied PDAC metastasis was measured. Exemplary histopathologic samples for the different tumor cellularity categories of the biopsy cylinders determined quantitatively (high: >67% (**A**); intermediate: 34–66% (**B**); low: <33% (**C**)) by a board-certified pathologist specialized in hepatic pathology are shown. (**D**) Attenuation in HU determined by automatic 3D segmentation of the biopsied metastasis normalized to the attenuation of aorta in HU and cellularity levels of the biopsy cylinders are shown. (**E**) Attenuation of the manual, single-slice, 2D-segmented metastasis normalized to the attenuation of the aorta and cellularity levels are shown. Black squares represent for each included patient the cellularity and normalized attenuation of the biopsied metastasis. No significant differences were found in one-way ANOVA (*p* = 0.88 for (**D**); *p* = 0.49 for (**E**)).

Different numbers of biopsy cylinders were available in the pathologic samples. In 50% (*n* = 18) of cases, three or more than three cylinders were available; in 22% (*n* = 8), three cylinders were available; in 19% (*n* = 7), two cylinders were available; and in only 8% (*n* = 3), one sample was available. The mean fraction of necrosis was 12%, ranging from 0% to 65% (Table 1 and Figure 3). While six (17%) patients had a low cellularity of less than 33%, 15 patients (42%) had a cellularity of 33–67%, and 15 (42%) a high cellularity (>67%) (Table 2 and Figure 3).

### 3.3. Differences in Attenuation between the Metastases of Different Cellularity Levels

In automatic 3D segmentation, the group with low cellularity had a normalized attenuation of 0.46 ± 0.10, the group with intermediate cellularity had one of 0.45 ± 0.10, and the group with high cellularity had one of 0.44 ± 0.13 (Table 2). In 2D manual segmentation, the low cellularity group had a normalized attenuation of 0.51 ± 0.10, the group with intermediate cellularity had one of 0.43 ± 0.12, and the group with high cellularity had one of 0.46 ± 0.17. We could not detect significant differences in the attenuation of the liver metastases between the groups with different cellularity levels, either for 3D segmentation (*p* = 0.88; Figure 3D) or for 2D segmentation (*p* = 0.49; Figure 3E).

### 3.4. Correlation between Attenuation and Fraction of Necrotic Tissue

**Figure 4:**
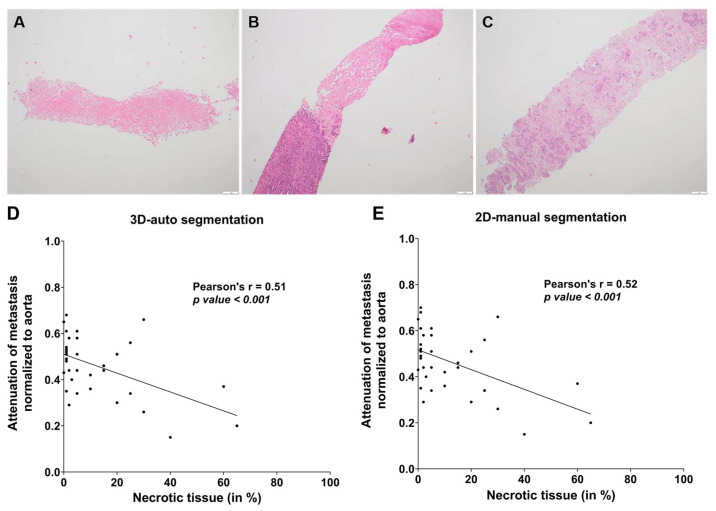
Correlation of attenuation with the necrosis fraction of PDAC metastases. Attenuation in Hounsfield Units of automatic 3D segmentation and manual 2D segmentation of the respective PDAC metastasis was measured. Fraction of necrosis in percent was scored quantitatively by a board-certified pathologist specialized in hepatic pathology. Exemplary samples with (**A**) high (≥ 20%), (**B**) intermediate (≥5% to <20%), and (**C**) low (<5%) necrosis. (**D**,**E**) Black dots represent for each included patient the fraction of necrosis and normalized attenuation of the biopsied metastasis. Pearson’s correlation between fraction of necrosis and attenuation of metastasis normalized to the aorta was performed for both segmentation modes.

The normalized attenuation of the automatic 3D-segmented metastasis showed a significant inverse-linear correlation with the fraction of necrotic tissue (Pearson’s r = 0.51, *p*-value < 0.001; Figure 4D). Interestingly, we could also confirm a significant inverse-linear correlation between the attenuation of the manual, single-slice, 2D-segmented metastasis and the fraction of necrotic tissue (Pearson’s r = 0.52, *p* < 0.001, Figure 4E) at a similar correlation coefficient.

### 3.5. Prognostic Impact of Tumor Necrosis of Hepatic Metastases

**Figure 5:**
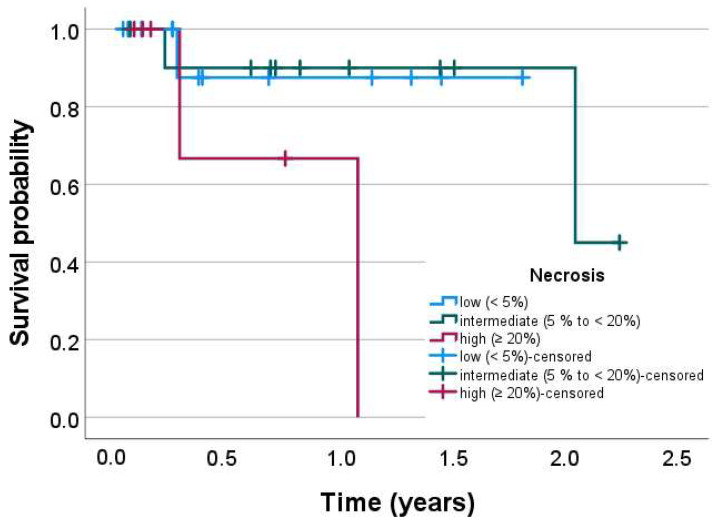
Prognostic impact of tumor necrosis fraction of hepatic metastases on the overall survival (OS) of therapy-naïve patients. Kaplan–Meier survival analysis was performed for the OS of all included patients (*n* = 31) without prior systemic therapy or metachronous appearance of metastases, as in these patients, the OS timespan was biased. OS was significantly different overall between the groups (*p* < 0.035) and in pairwise comparisons between the high- and medium-necrosis groups (*p* < 0.037) and the high- and low-necrosis (*p* < 0.033) groups, while there was no difference between the low and intermediate groups.

We performed Kaplan–Meier analyses for OS for the three pathologically determined necrosis levels (low: <5%; intermediate: ≥5 to <20%; high: ≥20%). Patients with metachronous metastases and systemic therapy prior to imaging (*n* = 5) were excluded. Patients with high necrosis fraction in hepatic metastases had a significantly worse survival probability (*p* < 0.035) than did patients with medium (*p* < 0.037) or low (*p* < 0.033) metastatic tumor necrosis (Figure 5).

## 4. Discussion

In this study, we examined the correlation between the attenuation of hepatic metastases of PDAC and their fraction of necrotic tissue and cellularity in venous-phase contrast-enhanced CT scans. We found a significant inverse correlation between the necrosis and attenuation of the metastases. In contrast, there was no correlation between the cellularity and the normalized attenuation. Additionally, we demonstrated metastatic necrosis to be negative prognostic biomarker in metastatic PDAC.

Identification of necrosis in hepatic metastases has clinical implications, as we found high necrosis to be a negative prognostic biomarker for OS in our cohort. Recently, other studies have focused on image-depicted tumor necrosis as a negative biomarker for resectable PDAC. A well-designed retrospective study identified primary tumor necrosis in CT imaging as an independent risk factor for recurrence or death in a risk model for recurrence-free survival [16]. Other recent studies have described primary tumor necrosis depicted by MR imaging as detrimental in terms of tumor size, nodal status and occurrence of metastases [9], recurrence-free survival, and OS [10,17].

Yet, our study is the first report of imaging used to identify necrosis in PDAC metastases and its prognostic impact. We validated venous-phase CT scans in quantifying metastatic necrosis via histopathologic comparison in patients without systemic therapy. Although we observed a significant correlation (*p* < 0.001), the correlation coefficient was only acceptable (Pearson’s r = 0.51), which may be explained by partial volume effects, as we compared image features of the whole metastasis volume to only a small part of the metastasis present in the biopsy cylinder.

An additional implication of findings may involve planning biopsy sampling patterns, as samples with a low necrosis ratio are desired for proper pathological workup. We demonstrated the potential of contrast-enhanced scans to identify vital lesions for biopsy in metastatic pancreatic cancer, which are the highest attenuating lesions. The significant correlation between attenuation and necrosis had an acceptable correlation coefficient if the full volume of the metastasis was segmented, which was consistent for segmentation in just a single slice (2D). This underlines the feasibility of this approach in clinical routine, as 2D segmentation is time- and resource-efficient.

We could not find a correlation between attenuation of the hepatic metastases of PDAC and cellularity. In contrast, one prior study reported a significant correlation between the attenuation of PDAC primary tumor and cellularity levels [14]. Likely, in the presence of tumor necrosis, intravoxel partial volume effects may mask true cellularity in primary tumors or metastases.

From our findings, one can expect that the presence of necrosis of hepatic metastases may serve as a negative prognostic finding. However, this hypothesis must be examined in further studies. In addition, normalized attenuation as a surrogate for tumor necrosis in hepatic metastases of PDAC may also be a useful parameter for evaluating tumor regression in systemic treatment approaches. Again, this hypothesis must be proven in further studies and is beyond the scope of the current project.

Our study has important limitations that need to be discussed. In general, the limited cohort size and the retrospective nature of our single-center study may reduce the generalizability of our results. Our study should be regarded as a proof-of-principle study and requires prospective validation in a multicenter study. Only one CT scan, performed on scanners of one manufacturer, was used in this study, which puts the external validity of our study into doubt, although it can be expected that the attenuation ratio is robust between different manufacturers.

In summary, the preliminary findings of our study may impact clinical case management, although prospective validation of the individual applications remains to be performed. CT estimation of metastatic necrosis at baseline examination might serve as prognostic biomarker in oncological risk models to influence therapy decisions. Furthermore, estimation of metastatic necrosis might serve as criterion for the radiologic evaluation of oncologic remission under systemic chemotherapy. Lastly, it may be helpful in biopsy pattern planning for CT-guided percutaneous biopsies.

## 5. Conclusions

We found a significant inverse correlation between the contrast-enhanced CT attenuation of hepatic metastases of PDAC and pathologic tumor necrosis. Additionally, there was a significantly shorter survival time in therapy-naïve patients with high metastatic necrosis levels. Pending prospective validation, our results could inform prognosis estimation, biopsy pattern planning, and the radiologic evaluation of oncologic follow-up imaging.

## Figures and Tables

**Table 1:** Clinical characteristics and histopathological features of the included patients.

Variable	Classes	N (%)
Sex	Male	17 (47%)
	Female	19 (53%)
Age	Mean (years)	65.5
	SD (years)	10.5
Grading of Metastasis	G1	0 (0%)
	G2	5 (14%)
	G3	3 (8%)
	NA	28 (78%)
Resection of primary tumor	yes	4 (11%)
	no	32 (89%)
Type of hepatic metastasis	Synchronous	32 (89%)
	Metachronous	4 (11%)
Number of biopsy cylinders	1	3 (8%)
	2	7 (19%)
	3	8 (23%)
	>3	18 (50%)
Fraction of necrosis	Mean	11.9%
	SD	16.2%
Tumor cellularity	Low (<33%)	6 (17%)
	Intermediate (33–67%)	15 (42%)
	High (>67%)	15 (42%)

**Table 2:** The patient group divided into subgroups according to the tumor cellularity level (low: <33%; medium: 33–67%; high: >67%).

	Tumor Cellularity	Low (*n* = 6)	Medium (*n* = 15)	High (*n* = 15)	*p* Value
Category					
**Sex**					
Male		4 (67%)	6 (40%)	7 (47%)	0.54
Female		2 (33%)	9 (60%)	8 (53%)	
Age (in years)		68.2 ± 6.7	64.4 ± 10.0	65.6 ± 12.3	0.77
Attenuation	3D auto segmentation	0.46 ± 0.10	0.45 ± 0.10	0.44 ± 0.13	0.88
	2D manual segmentation	0.51 ± 0.10	0.43 ± 0.12	0.46 ± 0.17	0.49

## Data Availability

The data presented in this study are available on reasonable request from the corresponding author. The data are not publicly available due to regulations on data safety of the Technical University of Munich.
